# Direct Species Identification in Positive Blood Culture Bottles From Patients With Hematologic Malignancies

**DOI:** 10.7759/cureus.59043

**Published:** 2024-04-26

**Authors:** Noriyuki Watanabe, Sachie Koyama, Tomoya Maeda, Haruka Karaushi, Yoshitada Taji, Yohei Kawasaki, Naoki Takahashi, Kotaro Mitsutake, Yasuhiro Ebihara

**Affiliations:** 1 Clinical Laboratory, Saitama Medical University International Medical Center, Saitama, JPN; 2 Department of Hemato-Oncology, Saitama Medical University International Medical Center, Saitama, JPN; 3 Department of Infectious Diseases and Infection Control, Saitama Medical University International Medical Center, Saitama, JPN; 4 Department of Biostatistics, Graduate School of Medicine, Saitama Medical University, Saitama, JPN; 5 Department of Laboratory Medicine, Saitama Medical University International Medical Center, Saitama, JPN

**Keywords:** central venous line, direct species identification, hematologic malignancy, bloodstream infection, blood culture

## Abstract

Background

In patients with hematologic malignancies, faster species identification is particularly important in the management of bloodstream infection because of their immunocompromised and neutropenic status. In the present study, we analyzed direct species identification in patients with hematologic malignancies, and the factors that might influence the results of species identification.

Methods

We performed direct species identification using a Sepsityper^®^ kit (Bruker Corporation, Billerica, Massachusetts, United States) and compared the results with a conventional method in patients with hematologic malignancies. Forty-five positive blood culture bottles containing single microorganisms from 37 patients were analyzed by matrix-assisted laser desorption/ionization-time of flight mass spectrometry (MALDI-TOF MS). And patients’ clinical data were compared between the groups with spectral scores at acceptable and unacceptable levels.

Results

Direct species identification correctly identified 42 of 45 isolates and three were misidentified. While 35 of 45 isolates showed a spectral score ≥1.7 (acceptable identification), 10 isolates had a spectral score <1.7 (unacceptable identification) including three misidentified isolates. The group with a spectral score ≥1.7 had significantly lower white blood cell (p<0.01), neutrophil (p<0.01), and platelet (p<0.01) counts in addition to more frequent central venous (CV) line insertion (p=0.01). Multivariate analysis revealed that pathogen type (gram-positive or negative) and CV line insertion were associated with spectral scores.

Conclusion

Direct species identification using the Sepsityper kit is an upcoming approach for blood culture bottles, which were flagged as positive even in patients with hematologic malignancies when the spectral score was ≥ 1.7. Our study also indicates that direct identification is more accurate in patients with CV lines, and may be less accurate when gram-positive bacteria are detected.

## Introduction

Patients with hematologic malignancies usually experience various severe infections during their treatments. This occurs because their underlying conditions or treatments predispose them to an immunocompromised status. Because many patients receive myeloablative chemotherapy with multiple anti-cancer drugs, white blood cell levels fall below 0.5 × 10^9^L, which lowers the ability of the body to defend against bacterial infections. Such infections include bloodstream infections (BSIs), which are severe conditions associated with elevated mortality. The gold standard for the diagnosis of BSI is blood culture. In general, empirical treatment with broad-spectrum antibiotics is initiated immediately because species identification (ID) may take 24 hours or longer from blood sample collection [1,2]. Faster species ID is particularly important because of their neutropenic and immunocompromised status.

Direct ID from positive blood culture bottles is an application of matrix-assisted laser desorption/ionization-time of flight mass spectrometry (MALDI-TOF MS). It can provide ID results in less than one hour using bacterial pellets directly purified from positive blood culture bottles [3]. Blood culture bottle media contain a variety of nonbacterial proteins, such as those derived from blood cells, and these may interfere with the interpretation of bacterial protein proteome profiles. Then, it is necessary for accurate ID to remove these host proteins by pretreatment substantially. In pretreatment protocols, Sepsityper® Kit (Bruker Corporation, Billerica, Massachusetts, United States) is involved in the lysis of blood cells, followed by centrifugation and washing [4] for MALDI-TOF MS-bases ID method [5].

Recently, we reported on direct species ID without culture, in which blood samples were processed with a Sepsityper kit (direct method), and direct species ID was performed by MALDI-TOF MS [6]. In that study, we demonstrated that species ID could be performed more quickly while providing results that were highly concordant with those of the conventional method of culturing colonies routinely used in our hospital. In the present study, we analyzed direct species ID in patients with hematologic malignancies and the factors that might influence the results of species ID to determine the validity of this system.

## Materials and methods

Study design

This retrospective single-center analysis was performed at Saitama Medical University International Medical Center, Hidaka, Japan. The study was approved by the Institutional Review Board of Saitama Medical University International Medical Center (approval number: #19-241) and conformed to the tenets of the Declaration of Helsinki. The need for informed consent was waived because of the retrospective nature of the study and the anonymity of the data.

We examined 45 blood samples flagged as positive from 37 patients with hematologic malignancies who were treated at the Department of Hemato-Oncology, Saitama Medical University International Medical Center, between December 2019 and March 2023. All data and clinical diagnoses were obtained from the patient records. Some patients had episodes of two or three positive blood cultures because the former episode had resolved before the next positive blood sample result.

Preparation of samples

Conventional and direct methods were performed at our hospital. When a BSI was suspected, blood was drawn and injected into blood culture bottles (BACTEC® Plus Aerobic/F, Anaerobic/F; Becton, Dickinson and Company, New Jersey, United States). These blood cultures were incubated using the BACTEC FX system (Becton, Dickinson and Company). In the conventional method, Gram stain and cultures were performed using these culture media when blood culture bottles were flagged as positive. After the Gram stain, species ID was performed with the microorganism grown on a 5% sheep blood agar medium (Kyokuto Pharmaceutical Industrial Co Ltd, Tokyo, Japan) and MacConkey agar medium (Eiken Chemical, Tokyo, Japan).

In the direct method, the samples were residual clinical specimens not collected specifically for this study. The direct method was performed within six hours after the conventional method. The Sepsityper kit was used according to the manufacturer’s instructions. Finally, a pellet was obtained after centrifugation and used as a sample for species ID.

Species ID analysis

The microorganisms cultured on agar in the conventional ID method and the pellets in the direct ID method using the Sepsityper kit were spotted onto an MSP 48 target polished steel BC plate (Bruker Corporation) and processed according to the manufacturer’s instructions. ID was performed by MALDI-TOF MS on a Microflex LT platform (MALDI Biotyper® Ver. 8.0; Bruker Corporation) according to the manufacturer’s instructions. Mass spectra were analyzed using Biotyper ver. 3.4 software and library (7854 isolates; Bruker Corporation).

In accordance with the manufacturer’s instructions, spectral scores ≥2.0 were accepted for species ID, and scores of 1.70-1.99 were accepted for genus ID; no ID was accepted for scores <1.7.

Measurement of biomarkers

Serum C-reactive protein (CRP) and total protein concentrations were evaluated by latex-enhanced turbidimetric immunoassay using CRP-Latex (II) X2 Seiken assay kit (Denka Seiken Co Ltd, Tokyo, Japan) and by Biuret method using Aqua-Auto Kinos TP-II (KAINOS Laboratories, Inc., Tokyo, Japan), respectively with a LABOSPECT 008 alpha automated analyzer (Hitachi High-Technologies Corporation, Tokyo, Japan). WBC, neutrophil, hemoglobin, and platelet were analyzed on the Sysmex XN-9100 (Sysmex Corporation, Kobe, Japan). These samples were measured at the same time or within 12 hours from the time that the blood culture was drawn.

Statistical analysis

Continuous variables are expressed as the median (Interquartile range (IQR)). Continuous variables were compared using the Mann-Whitney test. Fisher's exact test was used to compare categorical variables. Multivariate analysis was performed using logistic regression analysis. All statistical analyses were performed with EZR (Easy R) (Saitama Medical Center, Jichi Medical University, Saitama, Japan), which is a graphical user interface for R (The R Foundation for Statistical Computing, Vienna, Austria) [7]. Statistical significance was considered when p-value <0.05.

## Results

We examined 45 blood culture bottles from 37 patients that were flagged as positive. The patients’ profiles are shown in Table 1. The median age was 59 years (range, 18-86 years) and 27 patients were male (n=27; 73.0%). All patients had hematologic malignancies. Neutropenia (neutrophil count <0.5 × 10^9^/L) was found in 28 of the 45 blood culture bottles flagged as positive (62.2%). 

**Table 1:** Comparison of patients’ characteristics between acceptable and unacceptable identification by direct method Values are presented as n and median (IQR). WBC, neutrophils, platelet counts, hemoglobin (Hb), and CRP values were measured at the same time or within 12 hours from the time that blood culture was drawn. ALL: acute lymphoblastic leukemia; AML: acute myelogenous leukemia; NHL: non-Hodgkin lymphoma; WBC: white blood cell; Hb: hemoglobin; CRP: C-reactive protein; GPC: gram-positive cocci; GNR: gram-negative bacilli; CV: central venous catheter line; CRBSI: catheter-related bloodstream infection; CML: chronic myelogenous leukemia; MPAL: mixed phenotype acute leukemia; CLL: chronic lymphocytic leukemia); IQR: interquartile range

Characteristics	Total (N=45)	Spectral score		p-value
		<1.7 (unacceptable) (N=10)	≥1.7 (acceptable) (N=35)	
Age (years) at blood culture drawn, median (IQR)	59 (50-75)	66 (60-75)	56 (47-75)	0.104
Female/Male, n	10/27	2/8	7/28	1.00
Disease at blood culture drawn				0.413
ALL, n	5	0	5	
AML, n	17	5	12	
NHL, n	18	5	13	
Others, n	5	0	5	
WBC (×10^9^/L), median (IQR)	6.92 (0.09-6.29)	7.43 (2.54-14.69)	0.15 (0.07-4.78)	0.006
Neutrophil (×10^9^/L), median (IQR)	0.03 (0-4.65)	5.40 (0.47-6.90)	0.00 (0-1.45)	0.005
Neutropenia, n	28	3	25	0.027
Hb (g/dL), median (IQR)	9.4 (8.1-10.2)	9.5 (8.2-11.4)	9.3 (8.2-9.8)	0.575
Platelet (×10^9^/L), median (IQR)	22 (14-89)	81.5 (36.5-126.5)	19.0 (11.5-55.0)	0.008
CRP (mg/dL), median (IQR)	5.64 (1.42-10.71)	6.75 (1.79-14.25)	4.51 (1.58-10.09)	0.293
Total protein (g/dL), median (IQR)	5.8 (5.2-6.3)	5.85 (4.75-8.48)	5.90 (5.30-6.30)	0.682
Pathogen				0.147
Gram-positive, n	25	8	17	
Gram-negative, n	20	2	18	
CV insertion, n	26	2	24	0.010
CRBSI, n	3	2	1	0.119

Species ID

Forty-five isolates (25 gram-positive bacteria: 24 gram-positive cocci and one gram-positive bacilli, and 20 gram-negative bacteria: 20 gram-negative bacilli) were identified at spectral score ≥2.0 by conventional methods. Neither multiple microorganisms nor fungi were detected in the blood culture bottles. None of the isolates were identified as having “no peak” by the MALDI Biotyper.

Direct species ID correctly identified 42 of 45 isolates, and three were misidentified. While 35 of 45 isolates showed a spectral score ≥1.7 (acceptable ID), 10 isolates had a spectral score <1.7 (unacceptable ID) including three misidentified isolates.

A central venous (CV) line was inserted in 35 isolates identified from 26 patients. In these 26 patients, 35 episodes of BSI were found. More gram-positive bacteria (18 isolates) were detected in patients with a CV line insertion and more gram-negative bacteria (12 isolates) were detected in patients without a CV line. The distribution of detected pathogens was significantly different between patients with and without CV line insertion (p=0.039). There were no significant differences between the isolation of bacteria in patients with or without neutropenia (p=0.767).

In the ID of gram-positive bacteria, the direct ID method identified five out of 25 isolates (20.0%) with spectral scores ≥2.0 (species level) as well as 17 (68.0%) at the genus level (spectral scores ≥1.7) and eight (32.0%) at a non-acceptable level (spectral scores <1.7), as shown in Table 2.

**Table 2:** MALDI-TOF MS identification in the direct method (N=45) *These two isolates were misidentified by the direct method. **One of the isolates was misidentified by the direct method. MALDI-TOF MS: matrix-assisted laser desorption/ionization-time of flight mass spectrometry

Identification by convention method	Spectral score by direct method	
	<1.7 (unacceptable)	≥1.7 (acceptable)
Gram-positive bacteria		
Staphylococcus epidermidis	2	3
Staphylococcus aureus	0	2
Staphylococcus hominis	1*	0
Streptococcus mitis/oralis	1	6
Streptococcus agalactiae	0	1
Streptococcus dysgalactiae	1	0
Enterococcus faecium	1	2
Enterococcus faecalis	0	1
Parvimonas micra	2**	0
Kocria rhizophia	0	1
Corynebacterium jeikeium	0	1
Gram-negative bacteria		
Escherichia coli	0	9
Enterobacter cloacae complex	0	1
Klebsiella pneumoniae	0	4
Klebsiella oxytoca	0	1
Pseudomonas aeruginosa	0	2
Raoultella ornithinolytica	1	1
Stenotrophomonas maltophilia	1*	0

All 17 isolates with spectral scores ≥1.7 in the direct method were matched with the conventional method. Two isolates with spectral scores <1.7 did not match the ID results obtained using the conventional method. These two isolates were misidentified as *Candida albicans* and *Staphylococcus capitis* by direct method, which were identified as *Parvimonas micra* and *Staphylococcus hominis* by the conventional method, respectively. 

Three of eight *Staphylococcus* spp (37.5%) and two of nine* Streptococcus* spp (22.2%) were at non-acceptable levels (spectral scores <1.7). One of two *Staphylococcus aureus* was methicillin‐resistant *Staphylococcus aureus* (MRSA).

In the ID of gram-negative bacteria, the direct ID identified 14 of 20 isolates (70.0%) at the species level and 18 of 20 (90.0%) at the genus level, as shown in Table 2. All 18 isolates with spectral scores ≥1.7 in the direct method were matched with the conventional method. Two isolates (10.0%) were identified at a non-acceptable level and one isolate was misidentified as *Agromyces subbeticus* by the direct method, which was identified as *Stenotrophomonas maltophilia* by the conventional method.

Comparison of patients’ clinical data between the groups with spectral scores ≥1.7 and <1.7 

To search for factors that might explain the difference in spectral scores between the groups, we compared the patients’ clinical data between the groups with spectral scores ≥1.7 and <1.7. As shown in Table 1, the underlying disease, CRP, hemoglobin, and total protein values were not significantly different between the groups. However, the group with spectral score ≥1.7 showed significantly lower WBC (p=0.006), neutrophil (p=0.005), and platelet (p=0.008) counts and a higher proportion of CV line insertion (p=0.01) compared with the other group. The pathogens isolated in the group with spectral score <1.7 tended to be gram-positive (8 /10 isolates: 80.0%) although the proportions of gram-positive and gram-negative pathogens were not significantly different (p=0.147, Table 1).

We next examined the factors associated with the spectral score by multivariate analysis. Logistic regression analysis was performed using neutrophil counts, pathogen type (gram-positive or negative), and CV line insertion as explanatory variables. We found that CV line insertion (OR, 44.4; 95%CI 3.29-599.00) and pathogen type (OR, 0.0262; 95%CI 0.00178-0.383) were associated with spectral score (Table 3).

**Table 3:** Multivariate analysis of factors related to spectral score for species identification spectral score ≥1.7 vs <1.7 CV: central venous; VIF: variance inflation factor

Variable	Odds ratio	95% confidential interval	P value	Vif
Neutrophil	0.8960	0.774-1.060	0.208	1.070882
Gram-positive bacteria	0.0262	0.00178 -0.383	0.00782	1.818324
CV line insertion	44.4	3.29-599.00	0.00426	1.735546

## Discussion

International epidemiological work has shown that, in recent years, the predominant pathogens have shifted from gram-positive to gram-negative bacteria in patients with hematologic disease, especially malignancy [8,9]. Our study identified 25 gram-positive and 20 gram-negative bacteria in blood culture bottles taken from patients with hematologic disease. The predominance might depend on the region, hematologic malignancy distribution, treatment protocol, and supportive care. In our hospital, patients with hematopoietic malignancies are administered polymyxin B sulfate and itraconazole (ITCZ) or micafungin (MCFG) for antibacterial and antifungal prophylaxis, respectively. This prophylaxis might also influence the predominance.

The rate of matched ID between the conventional and direct methods was 100% at the genus level (spectral scores ≥1.7) for both gram-positive and gram-negative bacteria. However, for gram-positive bacteria, about one-third (eight cases) of IDs by the direct method had a spectral score <1.7 (Table 2). Although it might occur by chance, six of these eight gram-positive bacteria were matched with the conventional method. For the ID of gram-negative bacteria, two isolates were identified at a spectral score <1.7, and one pathogen was mismatched with the conventional method. The trend for a higher rate of ID at a spectral score ≥1.7 for gram-negative bacteria and a lower rate for gram-positive bacteria was reported previously but the analysis was not limited to patients with hematologic disease [4,6,10-13]. The lower rate of the accurate ID of gram-positive bacteria might be attributed to their more robust cell wall, smaller pellet formation due to slow growth [4,10], or high affinity for red blood cells [12]. Our study showed that five of eight *Staphylococcus* spp (62.5%) and seven of nine *Streptococcus* spp (77.8%) were at the genus level (spectral scores ≥1.7) as shown in Table 2. Although a similar trend was found in previous work [10-13], this is a critical issue for patients with hematologic malignancies because the detection rates of *Staphylococcus* spp and *Streptococcus* spp are relatively high [8-9]. Further improvements to the direct ID method are needed, particularly for gram-positive bacteria.

When we analyzed the factors that influenced the difference between spectral score ≥1.7 and <1.7 by direct method, bacteria with negative Gram stain results or patients with CV line insertion were connected with higher accuracy of direct species ID. As mentioned above, the characteristics of gram-positive bacteria might be attributed to this difference.

CV line insertion might be a risk factor for BSI, especially catheter-related BSIs (CRBSI). Indeed, it has been reported that gram-positive bacteria, particularly coagulase-negative *Staphylococcus* spp, remain the leading cause of CRBSI [14]. At least one set of blood cultures was drawn from the CV line, which might contain more pathogens when CRBSI has occurred, suggesting that blood cultures drawn from the CV line might contribute to more accurate species ID. In our study, more gram-positive bacteria (18 isolates) were detected in patients with a CV line insertion, and the distribution of detected pathogens was significantly different between patients with and without CV line insertion (p=0.039). However, only three CRBSI, which were diagnosed according to the criteria [15], and one out of three CRBSI caused by coagulase-negative *Staphylococcus* spp, showed spectral score ≥1.7 (1.74). 

The median neutrophil count was 0×10^9^/L (0-0.115) in patients with a CV line and 4.656×10^9^/L (0.035-7.769) in patients without a CV line. Neutrophil count significantly differed according to CV line status (p=0.00176). Neutrophil count was not associated with the difference in spectral score (score <1.7 or ≥1.7) in our multivariate analysis, but a lower neutrophil count might affect the accuracy of ID because neutrophils play an important role in eliminating pathogens. Our results indicated that species ID by conventional method might be necessary when gram-positive bacteria were found by Gram stain in blood culture bottles flagged as positive or the patients who did not have CV line insertion (Table 3). The direct method misidentified three isolates. All three patients did not have CV line insertion. One misidentification (*Agromyces subbeticus* by direct method) occurred when blasts in peripheral blood rapidly increased (blast 57.14×10^9^/L), and another (*Candida albicans* by direct method) occurred under the tumor lysis syndrome. These pathophysiological conditions might contribute to the misidentification by direct method. 

Our study has some limitations. First, the sample size was small, comprising just 45 blood isolates from 37 patients over a 39-month period. All the patients whose blood cultures were flagged as positive in the study duration were enrolled in this study. The sample size might influence the big fluctuations of the odds ratio in multivariate analysis. Second, there were 11 gram-positive and seven gram-negative species in this study, but other microorganisms can cause a BSI. Recent improvements in supportive care including antibacterial and antifungal prophylaxis have reduced the frequency of BSI. Third, neither multiple microorganisms nor fungi were detected in this study. We need to evaluate whether this species ID is suitable for other organisms such as fungi and multiple microorganisms. Also, we did not confirm that this result was applied to patients with malignancies other than hematologic. Finally, we did not adjust all the confounding factors which might include unknown factors to perform multivariate analyses. Further research is needed to check the reliability and clinical impact of direct ID.

## Conclusions

The direct species ID using the Sepsityper kit is an upcoming approach for blood culture bottles even in patients with hematologic malignancies when the spectral score is ≥ 1.7. We should bear in mind that direct ID might be associated with lower accuracy in patients with hematologic malignancies who do not have a CV line or when gram-positive bacteria are found by Gram stain at blood culture bottles flagged as positive. Further research is needed to check the reliability and clinical impact of direct ID.
